# Honey bee (*Apis mellifera*) colonies benefit from grassland/ pasture while bumble bee (*Bombus impatiens*) colonies in the same landscapes benefit from non-corn/soybean cropland

**DOI:** 10.1371/journal.pone.0257701

**Published:** 2021-09-20

**Authors:** Gabriela M. Quinlan, Meghan O. Milbrath, Clint R. V. Otto, Rufus Isaacs

**Affiliations:** 1 Department of Entomology, Michigan State University, East Lansing, MI, United States of America; 2 U.S. Geological Survey, Northern Prairie Wildlife Research Center, Jamestown, ND, United States of America; University of Illinois at Urbana-Champaign, UNITED STATES

## Abstract

Agriculturally important commercially managed pollinators including honey bees (*Apis mellifera* L., 1758) and bumble bees (*Bombus impatiens* Cresson, 1863) rely on the surrounding landscape to fulfill their dietary needs. A previous study in Europe demonstrated that managed honey bee foragers and unmanaged native bumble bee foragers are associated with different land uses. However, it is unclear how response to land use compares between managed honey bees and a managed native bumble bee species in the United States, where honey bees are an imported species. Furthermore, to our knowledge, no such direct comparisons of bee responses to land use have been made at the colony level. To better understand how two different social bees respond to variation in land use, we monitored the weights of *A*. *mellifera* and *B*. *impatiens* colonies placed in 12 apiaries across a range of land use in Michigan, United States in 2017. *Bombus impatiens* colonies gained more weight and produced more drones when surrounded by diverse agricultural land (i.e., non-corn/soybean cropland such as tree fruits and grapes), while honey bee colonies gained more weight when surrounded by more grassland/pasture land. These findings add to our understanding of how different bee species respond to agricultural landscapes, highlighting the need for further species-specific land use studies to inform tailored land management.

## Introduction

The European honey bee, *Apis mellifera* L., 1758, is the most economically important pollinator across the world [1], providing pollination to a wide range of food and forage crops. Recent developments in bee rearing have also made the common eastern bumble bee, *Bombus impatiens* Cresson, 1863, commercially available for crop pollination in eastern North America [2], and studies show *B*. *impatiens* to be more efficient at pollinating certain crops than honey bees [3, 4]. However, the health of honey bees [5] and population stability of many species of bumble bee [6, 7] are in jeopardy, due in part to insufficient access to suitable nutrition within their foraging range [8].

Bees rely on flowers to fulfill their dietary needs, and honey bees and bumble bees, as social generalist foragers, likely have broadly similar macro- and micro-nutrient dietary requirements [9, 10]. But, whether honey bees and bumble bees benefit from similar landscape composition is an area of debate. A study in the Northern Great Plains found that landscapes that support productive honey bee colonies also support more abundant and diverse native bee communities [11]. However, a study in Europe found that honey bees and unmanaged bumble bees were associated with different floral resources: honey bees were observed foraging more often on mass-blooming crops and bumble bees showed intermediate preference for both mass-blooming crops and semi-natural habitats [12]. Honey bees and bumble bees exhibit different foraging strategies [13–15] as well as key differences in their nutrient intake [16, 17] which could lead to different responses to the same landscapes. Understanding how different pollinators respond to landscape composition is important to designing pollinator conservation strategies, as currently in the United States, pollinator conservation is generally not designed to target specific guilds [18, 19]. Furthermore, comparing the landscape response of a managed species of bumble bee, such as *B*. *impatiens*, to the response from managed honey bee, *A*. *mellifera*, could provide greater insights into how landscapes influence the health of key managed bee species.

Monitoring differences in foraging activity alone may not be a sufficient metric by which to compare species’ response to landscape composition. All previous studies, of which we are aware, use monitoring techniques such as sweep netting and bowl traps to compare the effects of land use and landscape composition on different groups of bees. However, these techniques only capture forager visitation, potentially masking downstream effects on colony-level productivity and fitness. One such metric of colony-level productivity is colony weight. Change in colony weight, for both honey bees and bumble bees, is closely tied to the amount of stored resources (pollen and nectar) as well as adult and developing bees, and therefore a good metric of comparison. Bumble bee colony fitness may also be assessed by the number of reproductives produced, which include gynes (virgin queens) and male drones. Bumble bee colonies have an annual cycle in which reproductives are produced seasonally [20]. Honey bee colonies, conversely, are perennial and overwinter with a single queen. A honey bee queen can live several years, and therefore there is not an equivalent measure of reproductive output for honey bee colonies [21].

To compare the productivity of two key managed, social, generalist species (*A*. *mellifera* and *B*. *impatiens*) in the same landscapes, in this study, we 1) compare colony weight changes of honey bee and bumble bees located at the same apiary locations, 2) model the association between colony weight change throughout a growing season to the area of multiple land use categories (corn/soybeans, non-corn/soy crops, forage land, forests, and developed land), and 3) investigate the relationship between bumble bee colony gyne and drone production and area of land use categories. Because these two species are each generalists, we expect both species’ colonies will show similar patterns in weight change among apiaries and we also expect that weight gain and the production of bumble bee reproductives (gynes/ drones) will be positively correlated with the area of surrounding forage land.

## Materials and methods

### Site selection and land use quantification

**Fig 1:**
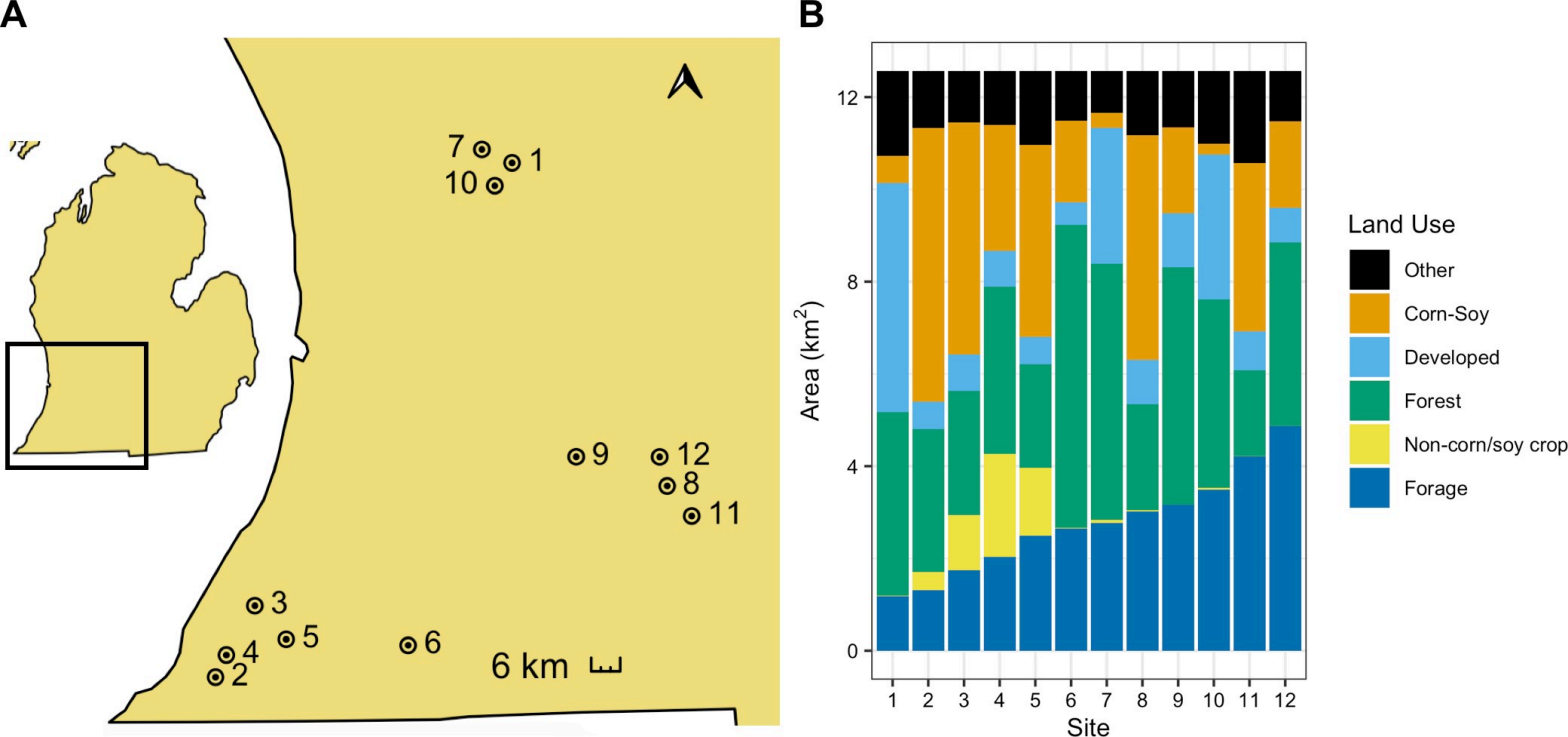
Location of 12 apiaries in Michigan, United States (A) and land use within 2 km (B) where honey bee and bumble bee colonies were sampled. Apiary locations are indicated with points, labeled with apiary number, and surrounded by a 2 km buffer. Land use is binned into six broad categories–forage, non-corn/soy crops, forests, developed, corn/soybeans, and all other land uses.

In the summer of 2017, we assessed weights of honey bee and bumble bee colonies at 12 apiary sites across southern Michigan. Apiary locations were selected from our collaborating beekeeper’s existing apiary locations to be spatially independent within a 2 km radius and to capture a range of land use available in the region (Fig 1A). Because these apiaries were on private land and the land owner provided us permission to access the colonies, field work permits were not required. Land use was determined using the 2017 30 m^2^ resolution Cropland Data Layer (CDL) [22]. In R Studio version 3.6.3 [23], the raster [24] and rgeos [25] packages were used to calculate the area of each land cover within 2 km of each focal apiary. These CDL land use classifications were then binned into six broad land use categories of interest: corn/soybeans, non-corn/soy crops (sugar beets, dry beans, potatoes, watermelons, cucumbers, peas, cherries, peaches, apples, grapes, asparagus, peppers, squash, blueberries, cabbage, celery), forage (sunflower, alfalfa, clover/wildflowers, other hay–non-alfalfa, fallow/idle cropland, grassland/pasture, woody wetlands, herbaceous wetlands, shrubland), forests (deciduous forest, mixed forest, evergreen forest), and developed land (developed open space, developed low intensity, developed medium intensity, developed high intensity) (S1 Table). An “other” (not applicable) category included grain crops (rye, oats, barley, spring wheat, etc.), other non-flowering crops (sod grass seed, Christmas trees), and undefined categories (other crop, barren).

### Bumble bee colonies

Three bumble bee colonies of *B*. *impatiens* (Koppert Biological Systems Inc., Howell, MI) were placed in each of the 12 apiary locations. Research colonies had one queen and approximately 50 workers and included a queen excluder to prevent gynes from exiting the colony when they were produced later in the season. Colonies were housed inside stacked milk crates with an insulated, reflective roof to minimize water, pest, and heat stress. On June 28, 2017, colonies were blocked by initial weight and placed in the field. Once a month from late-June to mid-August (June 28, July 24, August 10), colonies were weighed using a digital kitchen scale (AMIR Technology Co., Ltd., Shenzhen, CN). All foragers were inside the colony at the time of weighing: colony entrances were closed half-way the day before, which allowed returning foragers to enter but not leave. The colonies were removed from the field after drones (reproductive males) were observed exiting any colony (mid-August, approximately 6.5 weeks post-placement) and stored at -20°C until the number of gynes and drones (adults and pupae) were recorded [26]. By counting both adults and pupae, we captured the potential reproductive output of the colony, even if the colony was removed from the field before all reproductives reached maturity.

### Honey bee colonies

Each apiary contained on average 39 ± 2 (mean ± S.E.) commercial, migratory honey bee colonies. In each apiary, two honey bee colonies were placed on hive scales (SolutionBee, Raleigh, NC) that log colony weights every 15 minutes. Colonies were first inspected to ensure they were strong and contained a laying queen. Rather than use raw weights, which may be affected by the beekeeper adding or removing equipment, we instead calculated the cumulative change in weight over time for each colony. Sudden changes in colony weight (>3 kg/15 min) were removed, resulting in a smooth, continuous weight change curve [27]. We also filtered out data points in which the raw colony weight was less than 10 kg (approximate weight of an empty colony), which would suggest that the scale was malfunctioning or that the hive’s weight was not correctly distributed on the scale. After this data processing, honey bee colony weights (i.e., cumulative weight change) were collected on a similar schedule as the bumble bee colonies: June 30, July 24, and August 10. To obtain the most accurate weight, scales were read at midnight when all foragers should be inside the hive and when maintenance by the beekeeper is unlikely to occur.

### Statistical analysis

All statistical analysis was carried out in R Studio version 3.6.3 [23]. We assessed changes in weight gain over two time periods to accommodate potential differences in durations of colony growth between species [20, 21]: changes in colony weight were calculated for each colony between the first round (June 28/30) and third/final round (August 10), as well as between the first round (June 28/30) and second round (July 24). Within each apiary we averaged the change in weight of the two honey bee colonies and the change in weight and number of gynes and drones produced by the three bumble bee colonies. We also averaged honey bee and bumble bee weights within apiary and monthly weigh-in round. Due to hive scale malfunctioning, weight change values could not be calculated for four apiaries over the shorter time period (n = 8 remaining) and five apiaries over the longer time period (n = 7 remaining). Pearson’s product moment correlation was used to determine the correlation between the two species’ average colony weights and weight changes. Additive generalized linear models (GLM) were used to regress the change in weights and number of gynes and drones produced in each yard with the area of surrounding non-corn/soy crops, forage land, forests, and developed land, each scaled and centered. The area of corn/soybeans was strongly correlated (r = 0.68) with both forests and developed land and caused multicollinearity issues when included in the GLM. We chose to exclude the corn/soybean variable from our GLM because corn and soybeans are also not traditionally considered bee-supportive forage [28]. Once corn/ soybeans were excluded, none of the models had multicollinearity issues, based upon variance inflation factors (VIF < 3) [29]. The r2glmm package was used to calculate partial R^2^ values [30]. Because honey bees and bumble bees respond to landscapes at different spatial scales due to differences in their foraging ranges [31], we assessed each species’s response to land use over 1 km, 3 km, 4 km, and 6 km, in addition to our original range of 2 km (S2 Table). The area of each land use category was correlated (r > 0.40) across spatial scales, suggesting that landscape composition around our apiaries was relatively conserved across these distances (S3 Table).

## Results

**Fig 2:**
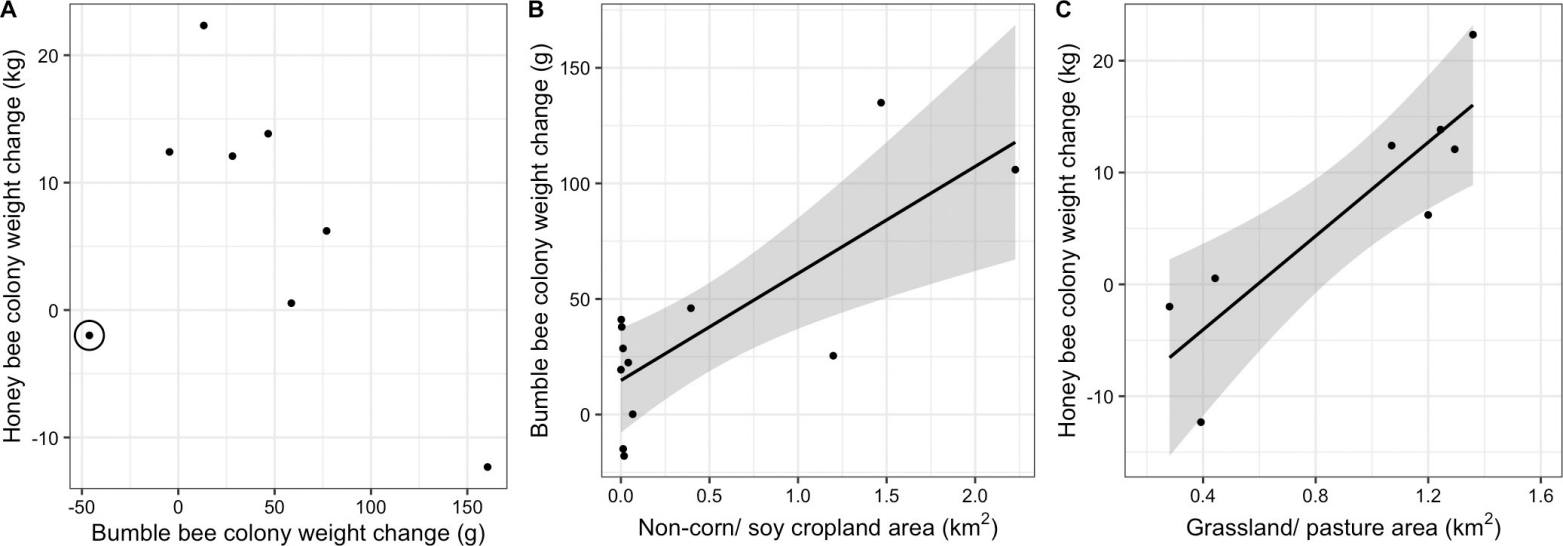
Correlation between bumble bee and honey bee colony weight change from late June to early August (A), area of non-corn/soy cropland within 2 km and change in bumble bee colony weight from late June to late July (B), and area of grassland/pasture within 2 km and change in honey bee colony weight from late June to early August (C), each in Michigan, United States in 2017. Points represent the average colony weight change within each apiary, with site 6 (Fig 2) circled in panel A. Without the inclusion of Site 6, which had the greatest proportion of forested land of all the sites, there is a significant correlation between bumble bee and honey bee colony weight change from late June to early August. Due to malfunctioning hive scales, only 8 of the 12 assessed apiaries are plotted for the honey bee weight change. The lines of best fit illustrate significant correlations (α< 0.05), and shaded region shows the confidence interval for the correlation.

Forests (primarily deciduous forests) were the dominant land use surrounding our apiaries, ranging from 15% - 52% of surrounding land area and averaging 30% of land within the 2 km surrounding area (Fig 1B). The area of non-corn/soy cropland (primarily tree fruits and grapes) ranged from 0% - 18% of the surrounding area and made up on average 4% of the surrounding area. Forage land ranged from 9% - 39% and averaged at 22% across sites and was made up, in large part, by woody wetlands and grassland/pasture. Our study area also included a range of corn/soy cropland (2% - 47%; 22% (min-max; mean)) and developed land (4% - 40%; 12%) (mostly open space and low intensity developed land) (Fig 2, S1 Table).

From late June to early August, honey bee colonies gained on average 6.64 kg ± 3.86 kg (mean ± S.E.), while bumble bee colonies gained on average 47.0 g ± 15.4 g. Over the shorter time period, from late June to late July, honey bees gained 5.48 kg ± 3.30 kg, while bumble bee colonies gained 35.8 g ± 13.0 g. Changes in weight of honey bee and bumble bee colonies kept in the same apiary were not significantly correlated to each other over either the shorter time period (r = -0.15, t_7_ = -0.39, p = 0.71) (weight change from June 28/30-July 24), or the longer time period (r = -0.50, t_6_ = -1.43, p = 0.20) (June 28/30-August 10) (Fig 2A). However, when apiary 6, which was surrounded by the greatest proportion of forested land (Fig 1B) was excluded, there was a strong negative correlation between honey bee and bumble bee weight change over the longer time period (r = -0.89, t_5_ = -4.32, p = 0.01) (Fig 2A). Honey bee and bumble bee colony weights were not correlated to each other at the first weigh-in in late June (r = 0.12, t_8_ = -0.33, p = 0.75), in late July (r = -0.28, t_8_ = -0.83, p = 0.43) or in early August (r = -0.39, t_8_ = -1.22, p = 0.26).

Bumble bee colonies surrounded by more non-corn/soy cropland gained significantly more weight in the shorter time period from late June to late July than bees with less non-corn/soy cropland (F_1,11_ = 13.36, partial-R^2^ = 0.58, p = 0.01) (Fig 2B). There was likewise a positive correlation for the longer time period, from late June to early August (F_1,11_ = 7.73, partial-R^2^ = 0.58, p = 0.03). Bumble bees also produced more drones when surrounded by more non-corn/soy cropland (F_1,11_ = 15.64, partial-R^2^ = 0.31, p = 0.01), but not more gynes (F_1,11_ = 0.11, partial-R^2^ = 0.19, p = 0.75). A greater number of gynes were produced in landscapes with greater amounts of forage land (F_1,11_ = 7.10, partial-R^2^ = 0.48, p = 0.03). Specifically, woody wetlands were positively correlated with gyne production (F_1,11_ = 8.65, R^2^_adj_ = 0.41, p = 0.01) when the primary forage land covers were de-aggregated and analyzed post-hoc as univariate GLMs. No other land use category (forage land, forests, or developed land) was significantly correlated with bumble bee colony weight change, drone production, or gyne production (S2 Table). Bumble bee colony weight change, drone production, and gyne production showed similar trends across spatial scales, with the strongest correlations between land use and colony outcomes occurring at shorter ranges, within the assessed 1km– 6 km range (S2 Table).

Conversely, honey bee colony weight change from late June to early August was negatively correlated with the amount of non-corn/soy cropland (F_1,7_ = 18.51, partial-R^2^ = 0.87, p = 0.02). This relationship was also negative for the shorter time period, from late June to late July but was not statistically significant (F_1,8_ = 1.49, partial-R^2^ = 0.11, p = 0.29). Honey bee colony weight change was also marginally negatively correlated with the area of surrounding forests over the longer time period (F_1,7_ = 110.97, parial-R^2^_adj_ = 0.78, p = 0.05). The negative correlations between non-corn/soy cropland and forests and weight change were consistent across spatial scales (S2 Table). While not significant, developed land showed a positive trend with honey bee colony weight gain across scales, and forage land showed a positive trend with colony weight gain at 6 km (S2 Table) and were thus explored further. Through post-hoc testing of the de-aggregated forage and developed land use classifications, we determined that honey bee colony weight change over the longer time period was positively correlated with grassland/ pastures (F_1,7_ = 19.66, R^2^_adj_ = 0.73, p<0.01) (Fig 2C), and this relationship held across spatial scales (S2 Table). However, the positive correlation was not statistically significant over the shorter time period (F_1,8_ = 5.24, R^2^_adj_ = 0.35, p = 0.06).

## Discussion

In this study we found differences in weight gain at the colony-level between native and non-native species of managed, social, generalist bees and also demonstrated divergent correlations with land use that could be driving these different responses. Our study helps advance national pollinator research goals by investigating the correlation between colony fitness surrogates (gyne and drone production) and land cover. Many bee habitat studies have inferred the importance of particular land covers by correlating bee abundance counts from monitoring with land covers (see [12, 32]). However, more research is needed to understand how bee demographic parameters (survival, fecundity, and movement) are affected by differences in land use.

Non-corn/soy cropland (primarily grapes and tree fruit) was associated with greater colony weight gain for *B*. *impatiens*, even though this landscape type made up a very small proportion of the surrounding area: most of our apiaries (8 out of 12 sites) had less than 3% of the surrounding area in this category, and the site with the greatest amount of non-corn/soy cropland had only 18%. Still, the trend between bumble bee colony weight gain and area of non-corn/soy cropland is compelling, given that the relationship is strongest at shorter foraging distances (where we would expect bumble bees to be spending the most time foraging [31]) and weakens as the radius increases. Overall, this finding was unexpected, as agricultural intensification in the midwestern United States has been proposed as a driver of reduced bumble bee diversity [33], and many bumble bee species have experienced declines in abundance and range constrictions over the last century [7, 33], due to a combination of stressors including habitat loss [34]. However, these same studies highlight that certain species, including *B*. *impatiens*, are thriving in their native range [7, 33]. Diet breadth may play an important role in determining the sensitivity of various bumble bee species to population decline, according to a correlational study [35]. Thus, a diversity of crops, such as those found in our study system, can support generalist foragers including *B*. *impatiens* [36]. In particular, diverse cropping systems provide complementary bloom pulses, that offer continuous forage availability [37, 38]. Weeds in field margins of non-corn/soy cropland may also provide important forage to bumble bees, even when crops are not in bloom [39]. The effect of diverse, non-corn/soy cropland is particularly pronounced in Michigan, which grows the second highest diversity of crops of all states in the United States [40]. Future studies aimed at capturing a greater range and a finer gradient of non-corn/soy cropland could greatly improve inference on the effect of this land use on bumble bee colony productivity.

Greater resource acquisition in these non-corn/soy agricultural landscapes may have promoted greater bumble bee drone production [41]. There was, however, no correlation between gyne production and non-corn/soy agricultural land in these colonies. A previous study found a similar positive correlation between landscape-scale resources and drone production but not gyne production [38]. Bumble bee colonies produce drones commensurate with resources, but gynes are more likely to be produced when resources are abundant (though the rate at which gynes are produced depends upon the bumble bee species) [41]. This could explain the lack of agreement in land-use associations between these castes. Instead, gyne production was marginally positively correlated with forage land, with which colony weight change and drone production showed a negative trend. This could represent a trade-off in reproductive investment by the colony [42] or could be a consequence of floral resource temporal availability [38]. Overall, previous findings on the effect of land use on bumble bee gyne production are mixed. Similar to our findings, a study in New York, United States found that *B*. *impatiens* colonies produced more gynes in more natural landscapes (forests, wetlands, pasture). This same study also found that agricultural landscapes (cucurbit, corn, soybeans) supported greater gyne production than suburban landscapes [43]. Conversely, a study in the United Kingdom found that *Bombus terrestris* colonies produced more gynes in urban and suburban landscapes but fewer gynes in more agricultural landscapes [44]. Further investigation into effects of diverse agricultural land (e.g., pesticide risk, nesting habitat, and disease pressure) on bumble bee reproductive outputs could enhance our understanding of the impact of agroecosystems on bumble bees.

Honey bee colony weight change was more strongly correlated with grassland/pasture. Grassland/pasture can support European plants (clovers and other non-native weeds), with which honey bees have a long association [45]. Furthermore, grassland/pastures likely have characteristics that support nectar gathering by honey bees. That is, honey bees are able to effectively recruit foragers to large sized parcels of land with abundant blooms [46, 47]. In a previous European study, honey bees were more associated with cropland, while bumble bees were associated with both semi-natural land and cropland [12], the opposite of what we observed. However, this difference likely stems from differences in habitat composition between studies (i.e., mass-blooming crops in the European system versus primarily tree fruits and grapes in our system). Michigan farms, on average, are small: less than half the size of the average United States farm [48], which may explain why honey bee colony weight gain was not positively associated with non-corn/soy cropland in our study, since small patches of land may not attract honey bee foragers. While we predicted that forage land would support colonies, the positive correlation between grassland/ pasture and colony weight change, specifically, was determined through post-hoc testing. Therefore, future studies that experimentally assess the effect of grassland/ pasture on honey bee colony weight gain may be warranted. Additionally, further analysis of the foraged pollen identity and the nectar rewards from plants in this region could help elucidate the relative importance of various plants and land use as sources of nutrition for the different bee species. We expect it is more likely that forage plants and habitat structure are driving differences in land use-associations between *A*. *mellifera* and *B*. *impatiens* through differences in foraging behavior [13–15], rather than interspecific competition. Unlike many states, Michigan does not require honey bee colony registration, making it difficult to determine local population density of honey bees. However, each research apiary contained approximately the same number of honey bee colonies within the apiary, and we have no reason to believe apiary locations would systematically differ in the number of colonies in the surrounding landscape.

Notably, neither bee species was positively correlated with the area of surrounding forested land. Forest is a natural land cover that is often included as bee habitat in large-scale models (see [49]). While forests provide early season forage as spring ephemerals and through woody plant bloom [50, 51], they likely provide very limited forage, especially for honey bees, in mid-to-late summer. In our study, the apiary surrounded by the most forested land had very low weight gain for both species. Assessing colony response to landscape at different times of the year could reveal colony-land use associations across temporal scales. Likewise, assessing these effects over multiple years to integrate the effects of weather could provide additional insights.

In this study we show divergent trends in colony weight change between two managed bee species associated with land use. Our findings, along with others [10, 52, 53] support the need for further research on species-specific resource requirements, to better understand the landscape-scale drivers of bee health and fitness. While pollinator species are generally likely to benefit from increased access to floral resources, our finding that two similar (generalist, social, managed) bee species, that are each relatively robust to population declines [33, 54], respond differently to the same types of land use highlights the need for further species-specific land use studies. Such insights could inform tailored land management to support healthy pollinator communities.

## Supporting information

S1 Table Area (km^2^) of land use within 2 km of each of the 12 honey bee and bumble bee apiary sites in Michigan, United States, based on the Cropland Data Layer (CDL) land use classification and the binned categories examined in this study. Land uses classified as “other” are not included in this table.
(DOCX)

S2 Table Correlations between land use (non-corn/soy, forage, corn/soybean, forests, and developed land) and bumble bee colony weight change from June 28/30-July 24, 2017, bumble bee colony gyne production, and honey bee colony weight change from June 28/30-August 10, 2017 across spatial scales (1 km, 2 km, 3 km, 4 km, 6 km) in 12 apiary sites in Michigan, United States. Effect estimate, degrees of freedom (DF), F-statistic, the adjusted R^2^ value, and p-value are provided for each model. Models significant at the α<0.05 level are indicated with a ‘*’ and those significant at α<0.10 are indicated with a ‘.’.
(DOCX)

S3 Table Correlation matrix of each land use (non-corn/soy, forage, corn/soybean, forests, and developed) across different distances (1 km, 2 km, 3 km, 4 km, 6 km) from 12 apiary sites in Michigan, United States. (DOCX)

S4 Table Bumble bee colony weight data. (CSV)

S5 Table Bumble bee colony gyne and drone production data. (CSV)

S6 Table Honey bee colony weight change data. (CSV)
