# Supplementary material for: Honey bee (Apis mellifera) colonies benefit from grassland/ pasture while bumble bee (Bombus impatiens) colonies in the same landscapes benefit from non-corn/soybean cropland
Source: PLoS One. 2021 Sep 20;16(9):e0257701. doi: 10.1371/journal.pone.0257701 (PMC8452077; doi:10.1371/journal.pone.0257701)
Supplement: S1 Table — Land uses classified as “other” are not included in this table. (DOCX) [file pone.0257701.s001.docx]

| Site | Binned Category | CDL Classification | Area (km^2^) |
| --- | --- | --- | --- |
| 1 | Non-Corn/ Soy Crop | Apples | 0.01 |
|  | Forage | Woody Wetlands | 0.72 |
|  |  | Grassland Pasture | 0.37 |
|  |  | Alfalfa | 0.07 |
|  |  | Herbaceous Wetlands | 0.01 |
|  |  | Shrubland | 0.01 |
|  | Corn/ Soy Crop | Corn | 0.19 |
|  |  | Soybeans | 0.39 |
|  | Forest | Deciduous Forest | 3.78 |
|  |  | Evergreen Forest | 0.18 |
|  |  | Mixed Forest | 0.01 |
|  | Developed | Developed Open Space | 3.34 |
|  |  | Developed Low Intensity | 1.37 |
|  |  | Developed Med Intensity | 0.26 |
|  |  | Developed High Intensity | 0.01 |
| 2 | Non-Corn/ Soy Crop | Apples | 0.14 |
|  |  | Grapes | 0.22 |
|  |  | Cherries | 0.02 |
|  |  | Blueberries | 0.01 |
|  | Forage | Woody Wetlands | 0.65 |
|  |  | Grassland Pasture | 0.19 |
|  |  | Alfalfa | 0.45 |
|  |  | Herbaceous Wetlands | 0.01 |
|  | Corn/ Soy Crop | Corn | 3.12 |
|  |  | Soybeans | 2.81 |
|  | Forest | Deciduous Forest | 3.07 |
|  |  | Evergreen Forest | 0.01 |
|  |  | Mixed Forest | 0.02 |
|  | Developed | Developed Open Space | 0.36 |
|  |  | Developed Low Intensity | 0.22 |
|  |  | Developed Med Intensity | 0.01 |
| 3 | Non-Corn/ Soy Crop | Apples | 0.29 |
|  |  | Grapes | 0.27 |
|  |  | Cherries | 0.42 |
|  |  | Cucumbers | 0.05 |
|  |  | Blueberries | 0.06 |
|  |  | Squash | 0.05 |
|  |  | Peppers | 0.06 |
|  |  | Celery | 0.01 |
|  | Forage | Woody Wetlands | 0.89 |
|  |  | Grassland Pasture | 0.44 |
|  |  | Alfalfa | 0.36 |
|  |  | Other Hay Non Alfalfa | 0.03 |
|  |  | Fallow Idle Cropland | 0.01 |
|  | Corn/ Soy Crop | Corn | 2.81 |
|  |  | Soybeans | 2.22 |
|  | Forest | Deciduous Forest | 2.68 |
|  |  | Evergreen Forest | 0.01 |
|  | Developed | Developed Open Space | 0.43 |
|  |  | Developed Low Intensity | 0.32 |
|  |  | Developed Med Intensity | 0.03 |
|  |  | Developed High Intensity | 0.01 |
| 4 | Non-Corn/ Soy Crop | Apples | 0.95 |
|  |  | Grapes | 1.04 |
|  |  | Cherries | 0.16 |
|  |  | Cucumbers | 0.02 |
|  |  | Blueberries | 0.01 |
|  |  | Peaches | 0.04 |
|  | Forage | Woody Wetlands | 1.10 |
|  |  | Grassland Pasture | 0.37 |
|  |  | Alfalfa | 0.52 |
|  |  | Other Hay Non Alfalfa | 0.01 |
|  |  | Fallow Idle Cropland | 0.03 |
|  |  | Shrubland | 0.01 |
|  | Corn/ Soy Crop | Corn | 1.20 |
|  |  | Soybeans | 1.53 |
|  | Forest | Deciduous Forest | 3.60 |
|  |  | Evergreen Forest | 0.01 |
|  |  | Mixed Forest | 0.01 |
|  | Developed | Developed Open Space | 0.56 |
|  |  | Developed Low Intensity | 0.22 |
| 5 | Non-Corn/ Soy Crop | Apples | 0.58 |
|  |  | Grapes | 0.39 |
|  |  | Cherries | 0.38 |
|  |  | Cucumbers | 0.05 |
|  |  | Blueberries | 0.03 |
|  |  | Squash | 0.01 |
|  |  | Peaches | 0.01 |
|  |  | Watermelons | 0.01 |
|  | Forage | Woody Wetlands | 1.62 |
|  |  | Grassland Pasture | 0.39 |
|  |  | Alfalfa | 0.45 |
|  |  | Other Hay Non Alfalfa | 0.03 |
|  |  | Herbaceous Wetlands | 0.01 |
|  | Corn/ Soy Crop | Corn | 2.19 |
|  |  | Soybeans | 1.97 |
|  | Forest | Deciduous Forest | 2.24 |
|  | Developed | Developed Open Space | 0.38 |
|  |  | Developed Low Intensity | 0.20 |
|  |  | Developed Med Intensity | 0.01 |
| 6 | Non-Corn/ Soy Crop | Grapes | 0.01 |
|  | Forage | Woody Wetlands | 1.76 |
|  |  | Grassland Pasture | 0.28 |
|  |  | Alfalfa | 0.54 |
|  |  | Herbaceous Wetlands | 0.01 |
|  |  | Clover Wildflowers | 0.04 |
|  | Corn/ Soy Crop | Corn | 0.85 |
|  |  | Soybeans | 0.92 |
|  | Forest | Deciduous Forest | 6.55 |
|  |  | Mixed Forest | 0.02 |
|  | Developed | Developed Open Space | 0.40 |
|  |  | Developed Low Intensity | 0.08 |
| 7 | Non-Corn/ Soy Crop | Apples | 0.01 |
|  |  | Cucumbers | 0.03 |
|  |  | Squash | 0.01 |
|  |  | Dry Beans | 0.01 |
|  | Forage | Woody Wetlands | 0.92 |
|  |  | Grassland Pasture | 1.61 |
|  |  | Alfalfa | 0.09 |
|  |  | Other Hay Non Alfalfa | 0.01 |
|  |  | Fallow Idle Cropland | 0.07 |
|  |  | Herbaceous Wetlands | 0.02 |
|  |  | Shrubland | 0.05 |
|  | Corn/ Soy Crop | Corn | 0.09 |
|  |  | Soybeans | 0.24 |
|  | Forest | Deciduous Forest | 5.38 |
|  |  | Evergreen Forest | 0.16 |
|  |  | Mixed Forest | 0.02 |
|  | Developed | Developed Open Space | 1.43 |
|  |  | Developed Low Intensity | 1.12 |
|  |  | Developed Med Intensity | 0.35 |
|  |  | Developed High Intensity | 0.04 |
| 8 | Non-Corn/ Soy Crop | Dry Beans | 0.01 |
|  | Forage | Woody Wetlands | 0.78 |
|  |  | Grassland Pasture | 1.36 |
|  |  | Alfalfa | 0.78 |
|  |  | Other Hay Non Alfalfa | 0.08 |
|  |  | Herbaceous Wetlands | 0.01 |
|  | Corn/ Soy Crop | Corn | 2.52 |
|  |  | Soybeans | 2.35 |
|  | Forest | Deciduous Forest | 2.29 |
|  |  | Evergreen Forest | 0.01 |
|  | Developed | Developed Open Space | 0.47 |
|  |  | Developed Low Intensity | 0.37 |
|  |  | Developed Med Intensity | 0.11 |
| 9 | Forage | Woody Wetlands | 1.49 |
|  |  | Grassland Pasture | 1.29 |
|  |  | Alfalfa | 0.38 |
|  | Corn/ Soy Crop | Corn | 1.51 |
|  |  | Soybeans | 0.35 |
|  | Forest | Deciduous Forest | 5.08 |
|  |  | Evergreen Forest | 0.05 |
|  |  | Mixed Forest | 0.01 |
|  | Developed | Developed Open Space | 1.04 |
|  |  | Developed Low Intensity | 0.13 |
|  |  | Developed Med Intensity | 0.01 |
| 10 | Non-Corn/ Soy Crop | Apples | 0.02 |
|  |  | Blueberries | 0.02 |
|  |  | Dry Beans | 0.01 |
|  | Forage | Woody Wetlands | 1.86 |
|  |  | Grassland Pasture | 1.24 |
|  |  | Alfalfa | 0.33 |
|  |  | Other Hay Non Alfalfa | 0.04 |
|  |  | Herbaceous Wetlands | 0.01 |
|  | Corn/ Soy Crop | Corn | 0.22 |
|  |  | Soybeans | 0.02 |
|  | Forest | Deciduous Forest | 3.95 |
|  |  | Evergreen Forest | 0.10 |
|  |  | Mixed Forest | 0.03 |
|  | Developed | Developed Open Space | 2.01 |
|  |  | Developed Low Intensity | 0.88 |
|  |  | Developed Med Intensity | 0.24 |
|  |  | Developed High Intensity | 0.01 |
| 11 | Forage | Woody Wetlands | 2.62 |
|  |  | Grassland Pasture | 1.07 |
|  |  | Alfalfa | 0.35 |
|  |  | Other Hay Non Alfalfa | 0.14 |
|  |  | Herbaceous Wetlands | 0.02 |
|  | Corn/ Soy Crop | Corn | 2.11 |
|  |  | Soybeans | 1.53 |
|  | Forest | Deciduous Forest | 1.85 |
|  | Developed | Developed Open Space | 0.62 |
|  |  | Developed Low Intensity | 0.19 |
|  |  | Developed Med Intensity | 0.03 |
|  |  | Developed High Intensity | 0.01 |
| 12 | Forage | Woody Wetlands | 3.11 |
|  |  | Grassland Pasture | 1.20 |
|  |  | Alfalfa | 0.51 |
|  |  | Other Hay Non Alfalfa | 0.03 |
|  |  | Herbaceous Wetlands | 0.01 |
|  |  | Shrubland | 0.01 |
|  | Corn/ Soy Crop | Corn | 1.03 |
|  |  | Soybeans | 0.85 |
|  | Forest | Deciduous Forest | 3.89 |
|  |  | Evergreen Forest | 0.08 |
|  | Developed | Developed Open Space | 0.62 |
|  |  | Developed Low Intensity | 0.11 |
|  |  | Developed Med Intensity | 0.03 |
