# Supplementary material for: Honey bee (Apis mellifera) colonies benefit from grassland/ pasture while bumble bee (Bombus impatiens) colonies in the same landscapes benefit from non-corn/soybean cropland
Source: PLoS One. 2021 Sep 20;16(9):e0257701. doi: 10.1371/journal.pone.0257701 (PMC8452077; doi:10.1371/journal.pone.0257701)
Supplement: S2 Table — Effect estimate, degrees of freedom (DF), F-statistic, the adjusted R2 value, and p-value are provided for each model. Models significant at the α<0.05 level are indicated with a ‘*’ and those significant at α<0.10 are indicated with a ‘.’. (DOCX) [file pone.0257701.s002.docx]

| Land use | Distance (km) | Estimate | DF | F-value | partial-R^2^ | p-value |
| --- | --- | --- | --- | --- | --- | --- |
| Bumble bee colony weight change (June 28/30 – July 24) | | | | | | |
| Non-corn/soy crop | 1 | 42.95 | 1,11 | 25.07 | 0.78 | <0.01* |
|  | 2 | 38.88 | 1,11 | 13.36 | 0.58 | <0.01* |
|  | 3 | 32.45 | 1,11 | 6.68 | 0.36 | 0.04* |
|  | 4 | 30.50 | 1,11 | 5.97 | 0.31 | 0.04* |
|  | 6 | 28.33 | 1,11 | 4.96 | 0.23 | 0.06. |
| Forage | 1 | 11.90 | 1,11 | 2.01 | 0.22 | 0.20 |
|  | 2 | 10.83 | 1,11 | 0.71 | 0.11 | 0.43 |
|  | 3 | 10.22 | 1,11 | 0.36 | 0.06 | 0.57 |
|  | 4 | 8.27 | 1,11 | 0.29 | 0.04 | 0.61 |
|  | 6 | 3.07 | 1,11 | 0.05 | 0.00 | 0.82 |
| Forest | 1 | -3.27 | 1,11 | 0.15 | 0.02 | 0.71 |
|  | 2 | -8.85 | 1,11 | 0.66 | 0.10 | 0.44 |
|  | 3 | -11.52 | 1,11 | 0.68 | 0.10 | 0.44 |
|  | 4 | -12.57 | 1,11 | 0.42 | 0.08 | 0.54 |
|  | 6 | -4.19 | 1,11 | 0.07 | 0.01 | 0.79 |
| Developed | 1 | 0.74 | 1,11 | 0.01 | 0.00 | 0.94 |
|  | 2 | 7.61 | 1,11 | 0.45 | 0.06 | 0.52 |
|  | 3 | 6.68 | 1,11 | 0.21 | 0.03 | 0.66 |
|  | 4 | 7.98 | 1,11 | 0.24 | 0.03 | 0.64 |
|  | 6 | 0.08 | 1,11 | 0.00 | 0.00 | 1.00 |
| Bumble bee drones | | | | | | |
| Non-corn/soy crop | 1 | 26.82 | 1,11 | 37.84 | 0.70 | <0.01* |
|  | 2 | 18.58 | 1,11 | 15.64 | 0.29 | 0.01* |
|  | 3 | 14.82 | 1,11 | 11.45 | 0.15 | 0.01* |
|  | 4 | 15.38 | 1,11 | 11.62 | 0.15 | 0.01* |
|  | 6 | 23.39 | 1,11 | 11.45 | 0.26 | 0.01* |
| Forage | 1 | -18.17 | 1,11 | 7.48 | 0.53 | 0.03* |
|  | 2 | -18.27 | 1,11 | 1.43 | 0.31 | 0.27 |
|  | 3 | -16.29 | 1,11 | 0.54 | 0.20 | 0.49 |
|  | 4 | -13.18 | 1,11 | 0.22 | 0.14 | 0.65 |
|  | 6 | -3.51 | 1,11 | 0.17 | 0.01 | 0.70 |
| Forest | 1 | -8.19 | 1,11 | 0.94 | 0.18 | 0.11 |
|  | 2 | -11.18 | 1,11 | 1.42 | 0.18 | 0.23 |
|  | 3 | -11.95 | 1,11 | 1.26 | 0.15 | 0.22 |
|  | 4 | -13.22 | 1,11 | 0.64 | 0.14 | 0.16 |
|  | 6 | -4.00 | 1,11 | 0.88 | 0.01 | 0.37 |
| Developed | 1 | -6.60 | 1,11 | 37.84 | 0.12 | 0.36 |
|  | 2 | -12.05 | 1,11 | 15.64 | 0.17 | 0.27 |
|  | 3 | -13.39 | 1,11 | 11.45 | 0.15 | 0.30 |
|  | 4 | -10.26 | 1,11 | 11.62 | 0.08 | 0.45 |
|  | 6 | -14.05 | 1,11 | 11.45 | 0.11 | 0.38 |
| Bumble bee gynes | | | | | | |
| Non-corn/soy crop | 1 | 5.78 | 1,11 | 0.15 | 0.21 | 0.71 |
|  | 2 | 6.91 | 1,11 | 0.11 | 0.19 | 0.75 |
|  | 3 | 6.12 | 1,11 | 0.01 | 0.11 | 0.93 |
|  | 4 | 5.02 | 1,11 | 0.00 | 0.07 | 0.99 |
|  | 6 | 2.10 | 1,11 | 0.01 | 0.01 | 0.94 |
| Forage | 1 | 12.78 | 1,11 | 10.09 | 0.58 | 0.02* |
|  | 2 | 12.97 | 1,11 | 7.10 | 0.48 | 0.03* |
|  | 3 | 12.29 | 1,11 | 4.61 | 0.38 | 0.07. |
|  | 4 | 10.43 | 1,11 | 2.93 | 0.27 | 0.13 |
|  | 6 | 5.56 | 1,11 | 0.36 | 0.06 | 0.57 |
| Forest | 1 | 0.37 | 1,11 | 3.42 | 0.00 | 0.89 |
|  | 2 | -1.49 | 1,11 | 0.09 | 0.02 | 0.78 |
|  | 3 | -2.32 | 1,11 | 0.15 | 0.03 | 0.71 |
|  | 4 | -2.29 | 1,11 | 0.05 | 0.02 | 0.83 |
|  | 6 | -3.93 | 1,11 | 0.09 | 0.03 | 0.77 |
| Developed | 1 | -2.33 | 1,11 | 44.95 | 0.04 | 0.61 |
|  | 2 | 2.44 | 1,11 | 0.24 | 0.03 | 0.64 |
|  | 3 | 2.68 | 1,11 | 0.22 | 0.03 | 0.66 |
|  | 4 | 2.35 | 1,11 | 0.12 | 0.02 | 0.74 |
|  | 6 | 3.61 | 1,11 | 0.17 | 0.02 | 0.69 |
| Honey bee colony weight change (June 28/30 – August 10) | | | | | | |
| Non-corn/soy crop | 1 | -11.78 | 1,7 | 16.81 | 0.85 | 0.03* |
|  | 2 | -17.11 | 1,7 | 18.51 | 0.87 | 0.02* |
|  | 3 | -9.81 | 1,7 | 3.18 | 0.42 | 0.17 |
|  | 4 | -7.16 | 1,7 | 2.62 | 0.28 | 0.20 |
|  | 6 | -4.37 | 1,7 | 3.41 | 0.15 | 0.16 |
| Forage | 1 | -2.37 | 1,7 | 0.31 | 0.25 | 0.62 |
|  | 2 | -5.71 | 1,7 | 0.27 | 0.53 | 0.64 |
|  | 3 | -1.83 | 1,7 | 0.20 | 0.04 | 0.69 |
|  | 4 | -0.06 | 1,7 | 0.12 | 0.00 | 0.76 |
|  | 6 | 4.14 | 1,7 | 0.19 | 0.12 | 0.70 |
| Grassland/pasture | 1 | 7.98 | 1,7 | 6.65 | 0.53 | 0.04* |
|  | 2 | 10.82 | 1,7 | 19.66 | 0.77 | <0.01* |
|  | 3 | 9.13 | 1,7 | 5.63 | 0.48 | 0.06. |
|  | 4 | 8.83 | 1,7 | 7.35 | 0.55 | 0.04* |
|  | 6 | 6.88 | 1,7 | 2.66 | 0.31 | 0.15 |
| Forest | 1 | -4.66 | 1,7 | 5.22 | 0.58 | 0.11 |
|  | 2 | -6.53 | 1,7 | 10.97 | 0.78 | 0.05* |
|  | 3 | -3.29 | 1,7 | 0.82 | 0.20 | 0.43 |
|  | 4 | -2.36 | 1,7 | 0.13 | 0.08 | 0.75 |
|  | 6 | -4.10 | 1,7 | 0.10 | 0.20 | 0.77 |
| Developed | 1 | 8.77 | 1,7 | 2.32 | 0.44 | 0.23 |
|  | 2 | 3.65 | 1,7 | 1.28 | 0.30 | 0.34 |
|  | 3 | 5.76 | 1,7 | 0.67 | 0.18 | 0.47 |
|  | 4 | 5.94 | 1,7 | 0.81 | 0.21 | 0.43 |
|  | 6 | 8.41 | 1,7 | 1.50 | 0.33 | 0.31 |
