# Supplementary material for: Honey bee (Apis mellifera) colonies benefit from grassland/ pasture while bumble bee (Bombus impatiens) colonies in the same landscapes benefit from non-corn/soybean cropland
Source: PLoS One. 2021 Sep 20;16(9):e0257701. doi: 10.1371/journal.pone.0257701 (PMC8452077; doi:10.1371/journal.pone.0257701)
Supplement: S3 Table — (DOCX) [file pone.0257701.s003.docx]

| Distance | 1 km | 2 km | 3 km | 4 km | 6 km |
| --- | --- | --- | --- | --- | --- |
|  | | | | | |
| Non-corn/soy crop | | | | | |
| 1 km | 1.00 | 0.97 | 0.91 | 0.89 | 0.88 |
| 2 km | 0.97 | 1.00 | 0.97 | 0.95 | 0.94 |
| 3 km | 0.91 | 0.97 | 1.00 | 1.00 | 0.99 |
| 4 km | 0.89 | 0.96 | 1.00 | 1.00 | 1.00 |
| 6 km | 0.88 | 0.94 | 0.99 | 1.00 | 1.00 |
|  | | | | | |
| Forage land | | | | | |
| 1 km | 1.00 | 0.85 | 0.71 | 0.65 | 0.40 |
| 2 km | 0.85 | 1.00 | 0.94 | 0.90 | 0.71 |
| 3 km | 0.71 | 0.94 | 1.00 | 0.99 | 0.88 |
| 4 km | 0.65 | 0.90 | 0.99 | 1.00 | 0.93 |
| 6 km | 0.40 | 0.71 | 0.88 | 0.93 | 1.00 |
|  | | | | | |
| Corn/soybean | | | | | |
| 1 km | 1.00 | 0.93 | 0.87 | 0.79 | 0.70 |
| 2 km | 0.93 | 1.00 | 0.98 | 0.93 | 0.86 |
| 3 km | 0.87 | 0.98 | 1.00 | 0.97 | 0.91 |
| 4 km | 0.79 | 0.93 | 0.97 | 1.00 | 0.96 |
| 6 km | 0.70 | 0.86 | 0.91 | 0.96 | 1.00 |
|  |  |  |  |  |  |
| Forest | | | | | |
| 1 km | 1.00 | 0.86 | 0.81 | 0.77 | 0.74 |
| 2 km | 0.86 | 1.00 | 0.95 | 0.85 | 0.76 |
| 3 km | 0.81 | 0.95 | 1.00 | 0.94 | 0.86 |
| 4 km | 0.77 | 0.85 | 0.94 | 1.00 | 0.97 |
| 6 km | 0.74 | 0.76 | 0.86 | 0.97 | 1.00 |
|  |  |  |  |  |  |
| Developed | | | | | |
| 1 km | 1.00 | 0.90 | 0.93 | 0.91 | 0.82 |
| 2 km | 0.90 | 1.00 | 0.98 | 0.92 | 0.83 |
| 3 km | 0.93 | 0.98 | 1.00 | 0.97 | 0.88 |
| 4 km | 0.91 | 0.92 | 0.97 | 1.00 | 0.95 |
| 6 km | 0.82 | 0.83 | 0.88 | 0.95 | 1.00 |
